# A case series of ten plus one deficiency of adenosine deaminase 2 (DADA2) patients in Iran

**DOI:** 10.1186/s12969-023-00838-3

**Published:** 2023-06-13

**Authors:** Kosar Asna Ashari, Nahid Aslani, Nima Parvaneh, Raheleh Assari, Morteza Heidari, Mohammadreza Fathi, Fatemeh Tahghighi Sharabian, Alireza Ronagh, Mohammad Shahrooei, Alireza Moafi, Nima Rezaei, Vahid Ziaee

**Affiliations:** 1 Pediatric Rheumatology Society of Iran, Tehran, Iran; 2 Children’s Medical Center, Pediatrics Center of Excellence, Tehran, Iran; 3 Department of Pediatrics, Tehran University of Medical Sciences, Tehran, Iran; 4 Pediatric Rheumatology Research Group, Rheumatology Research Center, Tehran University of Medical Sciences, Tehran, Iran; 5 Network of Immunity in Infection, Malignancy and Autoimmunity (NIIMA), Universal Scientific Education and Research Network (USERN), Tehran, Iran; 6 Department of Pediatrics, Isfahan University of Medical Sciences, Tehran, Iran; 7 Department of Pediatric Neurology, Pediatric Center of Excellence, Children’s Medical Center, Tehran, Iran; 8 Pediatric Rheumatology ward, Abuzar Children’s Hospital, Ahvaz Jundishapur University of Medica Sciences, Ahvaz, Iran; 9 Department of Pediatric Neurology, Alborz University of Medical Sciences, Karaj, Iran; 10 Department of Microbiology and Immunology, Laboratory of Clinical Bacteriology and Mycology, KU Leuven, Leuven, Belgium; 11 Research Center for Immunodeficiencies, Children’s Medical Center, Tehran University of Medical Sciences, Tehran, Iran; 12 Department of Immunology, School of Medicine, Tehran University of Medical Sciences, Tehran, Iran; 13 Division of Pediatric Rheumatology, Children’s Medical Center, No. 62 Dr. Gharib St., Keshavarz Blvd, Tehran, 14194 IR Iran

**Keywords:** Deficiency of adenosine deaminase 2, DADA2, Autoinflammatory syndrome, Livedo racemosa

## Abstract

**Background:**

Deficiency of adenosine deaminase 2 (DADA2) is an autosomal recessive autoinflammatory disease caused by mutations in the ADA2 gene. DADA2 has a broad spectrum of clinical presentations. Apart from systemic manifestations, we can categorize most of the signs and symptoms of DADA2 into the three groups of vasculitis, hematologic abnormalities, and immunologic dysregulations. The most dominant vasculitis features are skin manifestations, mostly in the form of livedo racemosa/reticularis, and early onset ischemic or hemorrhagic strokes. Hypogammaglobulinemia that is found in many cases of DADA2 brings immunodeficiencies into the differential diagnosis. Cytopenia, pure red cell aplasia (PRCA), and bone marrow failure (BMF) are the hematologic abnormalities commonly found in DADA.

**Case presentation:**

We introduce eleven patients with DADA2 diagnosis, including two brothers and sisters, one set of twin sisters, and one father and his daughter and son. Ten patients (91%) had consanguineous parents. All the patients manifested livedo racemose/reticularis. Ten patients (91%) reported febrile episodes, and seven (64%) had experienced strokes. Only one patient had hypertension. Two of the patients (11%) presented decreased immunoglobulin levels. One of the patients presented with PRCA. Except for the PRCA patient with G321E mutation, all of our patients delivered G47R mutation, the most common mutation in DADA2 patients. Except for one patient who unfortunately passed away before the diagnosis was made and proper treatment was initiated, the other patients’ symptoms are currently controlled; two of the patients presented with mild symptoms and are now being treated with colchicine, and the eight others responded well to anti-TNFs. The PRCA patient still suffers from hematologic abnormalities and is a candidate for a bone marrow transplant.

**Conclusions:**

Considering the manifestations and the differential diagnoses, DADA2 is not merely a rheumatologic disease, and introducing this disease to hematologists, neurologists, and immunologists is mandatory to initiate prompt and proper treatment. The efficacy of anti-TNFs in resolving the symptoms of DADA2 patients have been proven, but not for those with hematologic manifestations. Similarly, they were effective in controlling the symptoms of our cohort of patients, except for the one patient with cytopenia.

**Supplementary Information:**

The online version contains supplementary material available at 10.1186/s12969-023-00838-3.

## Introduction

The autoinflammatory disorder of the deficiency of adenosine deaminase 2 (ADA2) or DADA2 was first described by Zhou et al. in 2014. They found autosomal recessively inherited mutations in CECR1 (cat eye syndrome chromosome region, candidate 1) in nine patients [1]. The CECR1 gene, now known as the ADA2 gene, is mapped to chromosome 22q11.1. The mutation can cause a loss of function in the ADA2 protein, leading to reduced plasma activity of ADA2 [1].

ADA2 enzyme is secreted mainly by monocytes and cells of the myeloid lineage [2, 3]. Similar to ADA1, ADA2 has an enzymatic effect of catalyzing the deamination of adenosine and 2’-deoxyadenosine into inosine and deoxyinosine, respectively. Moreover, ADA2 is active in sites of inflammation, hypoxia, and tumor growth, where adenosine concentration is elevated, and the pH is acidic [2]. ADA2 has multiple roles, namely a growth factor-like action [2, 4], the autocrine activity of differentiating monocytes to macrophages [5], the interplay function between monocytes and T cells [3], and the balancing role between pro-inflammatory and anti-inflammatory states [6].

DADA2 has a broad spectrum of clinical presentations. Apart from systemic manifestations, most of the signs and symptoms of DADA2 can be categorized under the three groups of vasculitis, hematologic abnormalities, and immunologic dysregulation. The most dominant vasculitis features are skin manifestations, mainly in the form of livedo racemosa/reticularis, and early onset ischemic or hemorrhagic strokes. Hypogammaglobulinemia found in many cases of DADA2 brings immunodeficiencies into the differential diagnosis. Cytopenia, pure red cell aplasia (PRCA), and bone marrow failure (BMF) are the hematologic abnormalities commonly found in DADA2 [7–10].

Some vasculitis disorders mimic presentations of DADA2, such as polyarteritis nodosa (PAN), immunodeficiencies such as severe combined immunodeficiency (SCID) and autoimmune lymphoproliferative syndrome (ALPS), and hematologic disorders such as Diamond Blackfan anemia (DBA). Interestingly enough, DADA2 patients might present with autoimmune presentations like those of systemic lupus erythematosus (SLE) [11]. We now believe that many patients with this pathology had been misdiagnosed with another disorder which might have led to catastrophic consequences, a fact that makes presenting case series of DADA2 patients invaluable. Considering the diverse manifestations, a DADA2 patient might first be referred to a neurologist, hematologist, immunologist, or rheumatologist. That is why increased awareness of the characteristics of this disease is of utmost importance. Herein we present eleven cases of DADA2 that were diagnosed and treated in Iran, one of which presented initially with hematologic manifestations.

## Case presentations

We introduce eleven patients with DADA2 diagnosis including one brother and sister (patients seven and six), one set of twin sisters (patients four and five), and one father and his daughter and son (patients eleven, one, and two, respectively).

Seven patients (63%) were females. The range for age of onset was 0.5 to 13 years, median = 3 years. It is noteworthy that we did not know the exact age of onset of one of the patients who was an adult at the time of recruitment, but he expressed that the episodes had begun since childhood. The range for age of diagnosis was 3 to 37 years, median = 8 years. Ten patients (91%) had consanguineous parents. All the patients manifested livedo racemosa/reticularis. Ten patients (91%) reported febrile episodes, and seven (64%) had experienced strokes. Only one patient had hypertension. Nine patients (81%) had elevated inflammatory markers (erythrocyte sedimentation rate (ESR) and C-reactive protein (CRP)) when they were admitted to the hospital. Two patients (11%) presented with decreased immunoglobulin levels. ADA2 activity cannot be tested in Iran, and it is costly to send the specimen abroad and do the test. Therefore, it was only tested for two patients (patients 6 and 7), and the levels were zero for both. The clinical characteristics of the patients are summarized in Table 1.

One of the patients (patient eight) presented with neutropenia and severe anemia at four months. Bone marrow aspiration found PRCA. At the age of four years, hypopigmented skin lesions were found on the limbs, and the parents complained of periodic fevers accompanied by abdominal pain and oral aphthous ulcers. Autoantibodies were within normal limits. Since neutropenia persisted, granulocyte-colony stimulating factor (GCSF) was prescribed. She was later admitted with an episode of stroke. Laboratory evaluations revealed normal antinuclear antibody (ANA), anti-double-stranded (ds)DNA, perinuclear anti-neutrophil cytoplasmic antibodies (p-ANCA), cytoplasmic-antineutrophil cytoplasmic antibodies(c-ANCA), and lupus anticoagulant levels. C3, C4, CH50, and CD flow cytometry were normal. She had slightly elevated Dihydrorhodamine (DHR), decreased immunoglobulin (Ig)A and IgG levels, normal IgE level, and normal tetanus and diphtheria antibody levels. With hypersignal lesions found in the occipital region and hypodensity in the parietal lobe in magnetic resonance imaging (MRI), she was diagnosed with posterior reversible encephalopathy syndrome (PRES). At this stage, whole exome sequencing (WES) results were prepared, showing an autosomal recessive (AR) homozygous mutation in ADA2: p.G321E [rs865858930]. Sanger sequencing confirmed that the patient was homozygous while her parents were carrier heterozygous for this mutation.

**Fig. 1:**
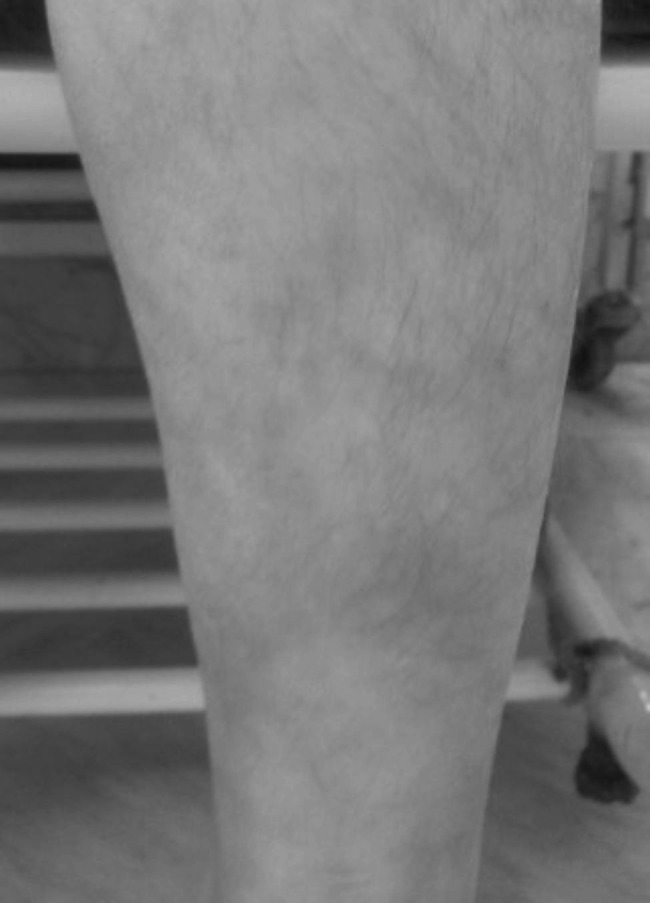
Livedo racemosa on the leg of patient one, a deficiency of adenosine deaminase 2(DADA2) patient

Patient one presented at five years with an episode of stroke, livedo racemosa, and increased inflammatory marker levels (Fig. 1). She was primarily diagnosed with neuro-Behcet’s disease, since her father (patient 11) had been diagnosed with Behcet’s disease and she had positive HLA B5 and HLA B51. She was being treated with methylprednisolone pulse and azathioprine. She continued to present episodes of stroke with evidence of hemorrhagic components and vascular nature on MRI. Laboratory evaluations showed elevated platelets, ESR, CRP, and negative c-ANCA, p-ANCA, anti-dsDNA, and ANA levels. Normal protein C, protein S, factor v, and lupus anticoagulant levels were reported. Antiphospholipid (APS) IgM antibody level was 2 g/L (normal range: 0.4–2 g/L), and IgG level was 67 g/L (normal range: 6–16 g/L). She had normal creatinine levels and normal renal sonography. At this point, PAN seemed to be the most suitable diagnosis, but could not explain all the symptoms. The blood pressure was consistently normal. Infliximab was prescribed, the fever stopped, and hemiparesis was improved. Heparin was initiated and then was changed into warfarin. She was discharged with aspirin (due to a positive APS antibody), prednisolone, and mycophenolate mofetil. At the age of 10, the patient had an episode of stroke, after which she had loss of consciousness and unfortunately passed away. After her demise, genetic analysis was ready, finding a missense homozygous mutation in G47R, compatible with DADA2. Interestingly, the patient’s brother was also experiencing episodes of fever, oral aphthous, and livedo racemosa, and her father had manifested fever, livedo racemosa, headaches, and myalgia/arthralgia since childhood. Upon genetic testing, the same homozygote mutation was found in the brother and father (patients 2 and 11). Since this patient was our only patient who, unfortunately, passed away, we discussed the characteristics in detail to understand the possible difference with other patients that could have led to the mortality.

Except for the PRCA patient with G321E mutation, all of our patients presented G47R mutation, the most common mutation in DADA2 patients. The mutations are mentioned in Table 1.

Out of the eleven patients, six are now under treatment with Etanercept and two with Adalimumab with favorable results. One of the patients (patient one) unfortunately passed away before having the chance to be treated with anti-TNFs. She was only very briefly treated with Infliximab which was discontinued since we did not know the diagnosis at that time. The brother of this patient (patient 2), presented with episodes of fever, oral aphthosis, and livedo racemosa. WES was done at the same time that it had been done for his sister, and he was diagnosed with DADA2 at the age of three. He has been treated with colchicine since the beginning of presentations and his attacks have not recurred in the last two years. Since he became symptom-free after the initiation of colchicine, the parents refused to initiate an anti-TNF therapy when DADA2 was diagnosed. For the same reason, the diagnosis of DADA2 for the father (patient 11) did not change the fact that he was being treated with colchicine. The patient with bone marrow failure (patient 8) was treated with anti-tumor necrosis factor (TNF), aspirin, and prednisolone. A few months after the initiation of anti-TNF, fever was controlled, and anti-inflammatory markers were within normal limits; however, anemia and neutropenia persisted. She is a hemopoietic stem cell transplantation (HSCT) candidate now.

**Table 1:** Characteristics of DADA2 patients. PRCA: pure red cell aplasia; Ig: immunoglobulin; het: heterozygous; hom: homozygous; GCSF: granulocyte-colony stimulating factor; IVIG: intravenous immunoglobulin; HSCT: hemopoietic stem cell transplantation, TNF: tumor necrosis factor

	Patient 1	Patient 2	Patient 3	Patient 4	Patient 5	Patient 6	Patient 7	Patient 8	Patient 9	Patient 10	Patient 11
**Gender**	F	M	F	F	F	F	M	F	F	M	M
**Consanguinity**	Yes	Yes	Yes	Yes	Yes	Yes	Yes	Yes	Yes	No	Yes
**Age at onset, yrs**	5.5	2.5	3	3	3	13	5	0.5	2	1.5	?
**Age at diagnosis, yrs**	10	3	9	6	6	16	6	8	3	9.5	37
**Current age, yrs**	-	6	12	9	9	17	7	9	3.5	10	40
**Fever**	Yes	Yes	Yes	Yes	Yes	Yes	Yes	Yes	No	Yes	Yes
**Skin findings**	Livedo racemosa	Livedo racemosa	Livedo racemosa	Bruising, livedo racemosa	Bruising, livedo racemosa	Livedo racemosa	Livedo racemosa	Hypopigmented lesions, livedo racemosa	Livedo racemosa	Livedo racemosa	Livedo racemosa
**Hematologic**	No	No	No	No	No	No	No	PRCA	Anemia	No	No
**Myalgia/arthralgia**	Yes	No	Yes	Yes	Yes	No	No	No	No	No	Yes
**Neurologic**	Recurrent strokes	No	Recurrent strokes	No	No	Stroke	Stroke	Stroke	Recurrent strokes	Stroke	Headaches
**Immunological**	No	No	No	No	No	No	No	Decreased IgA and IgG	No	No	Low IgM
**Renal**	No	No	Hypertension	No	No	No	No	No	No	No	No
**Other findings**	Oral aphthosis	Oral aphthosis	Genital ulcer	No	No	No	No	Oral aphthosis, abdominal pain	No	No	Oral aphthosis
**ADA2 gene mutation**	G47R Missense Hom	G47R Missense Hom	G47R Missense Hom	G47R Missense Hom	G47R Missense Hom	G47R Missense Het	G47R Missense Het	G321E Missense Hom	G47R Missense Hom	G47R Missense Hom	G47R Missense Hom
**Previous treatment**	Azathioprine, methylprednisolone pulse therapy, infliximab, heparin, warfarin, aspirin, mycophenolate mofetil, nifedipine	Colchicine	Methylprednisolone pulse, cyclophosphamide, propranolol, nifedipine, azathioprine, aspirin, prednisolone, diltiazem, infliximab	Prednisolone	Prednisolone	Aspirin, warfarin	-	GCSF, IVIG, methylprednisolone	-	-	-
**Current treatment**	-	Colchicine	Adalimumab	Etanercept	Etanercept	Etanercept	Etanercept	Etanercept, aspirin, prednisolone, HSCT candidate	Adalimumab	Etanercept Prednisolone	Colchicine
**Duration of treatment with anti-TNF (months)**	-	-	23	36	36	8	7	12	12	6	-
**Outcome**	Demise	Favorable, no sign or symptom	Favorable, only Raynaud remained	Favorable, no sign or symptom	Favorable, no sign or symptom	Favorable, no sign or symptom	Favorable, no sign or symptom	Persistence of anemia and neutropenia	Favorable, no sign or symptom	Favorable, no sign or symptom	Favorable, no sign or symptom

## Discussion

We have presented eleven patients with the diagnosis of DADA2 confirmed by their genetic mutations. DADA2 patients can present with a wide range of manifestations. Episodes of fever and systemic symptoms, vasculitis features such as the notable sign of recurrent strokes, skin manifestations (most commonly livedo racemosa/reticularis), hypertension, hematologic manifestations, and immunologic dysregulations are among the most common signs and symptoms. Besides the mentioned symptoms, our patients manifested oral ulcers, genital ulcers, myalgia, and headache, which are also reported in the literature, albeit less commonly [10, 12–14].

Lee et al. suggested categorizing DADA2 patients into three major groups: vasculitis, bone marrow failure (BMF), and PRCA [15]. They performed a literature review and found 100 cases of vasculitis, the most common presentation of DADA2, and 52 cases of BMF or PRCA. They also introduced an international cohort of five PRCA and ten BMF patients. Lee et al. found significant differences between the three groups. Analogous to interpretations of their cohort and the literature review, we reported one patient with PRCA phenotype. The number of DADA2 cases with hematologic abnormalities reported in the literature is much less than those with vasculitis, and we also found only one patient among 11 patients with this phenotype. Similar to most of the PRCA patients reported in the case series, our patient’s symptoms developed very early, at the age of four months. Her initial presentation was cytopenia, and she later developed vasculitis in the form of a stroke episode [15–17]. In this case series, we depicted the characteristics of our patient with PRCA phenotype in more detail, since we meant to point out the differences between DADA2 patients with mere vasculitis features and those who carry hematologic abnormalities. We divided our patients into groups of PAN-like patients and DBA-like patients, close to what LEE et al. have performed. The clinical characteristics of our two groups of patients are summarized in Table 2.

Interestingly enough, the presentations of DADA2 are not restricted to the three mentioned categories. Lupus-like manifestations have been reported in these patients [11]. Moreover, characteristics of immunodeficiencies in terms of cell depletions and antibody deficiencies can be found in DADA2 patients [18]. Considering the manifestations and the differential diagnoses, DADA2 is not merely a rheumatologic disease, and introducing this disease to hematologists, neurologists, and immunologists is mandatory to initiate prompt and proper treatment. It should be noted, however, that rheumatologic diseases can also be termed “systemic disorders”, meaning that a rheumatologist must have expertise in detecting any abnormality in any organ system. The treatment and follow-up of DADA2 patients can be managed best by a rheumatologist that should use a multi-disciplinary team for help. On the other hand, rheumatologists should try to raise awareness among pediatricians, internists, hematologists, and neurologists about the manifestations of this disease and the necessity of referral. A patient with stroke, for instance, should be systematically investigated by a neurologist and, if suspicious manifestations of DADA2 are found, referred to a rheumatologist as the team manager. It should be drawn into attention that the patients of this cohort were referred from doctors with different specialties or subspecialties, but the final diagnosis in all the cases was made by a rheumatologist.

ADA2 enzyme is a protein encoded by the ADA2 gene and is composed of four domains: signal peptide, a dimerization domain, catalytic domain, and putative receptor binding domain. The most common disease variant, p.Gly47Arg (p.G47R), is located in the dimerization domain [13, 19]. Ozen et al. surveyed the clinical and molecular differences between two groups of DADA2 patients: 14 in the PAN-like group and 10 in the DBA-like group. They reported that the catalytic domain of ADA2 was affected in the DBA-like patients, while the dimerization domain was affected in the PAN-like patients [10]. Similar to the study of Ozen et al., we can categorize our patients into PAN-like, who presented with vasculitis features and included eight patients, and DBA-like, which is our one patient presenting with anemia and neutropenia. All the patients in the PAN-like group had G47R mutation, and the one DBA-like patient had a p.Gly321Glu (P.G321E) mutation, located on the catalytic domain, resonating with the finding of Ozen et al. This genotype-phenotype correlation has convinced us to name our cohort as “ten plus one DADA2 patients”.

The mutations found in DADA patients are less commonly heterozygous. However, no correlation has been found between heterozygosity and the phenotype [10, 13]. Likewise, our two patients with heterozygous mutations of G47R, who were brother and sister, did not show distinct manifestations in comparison with the rest of the patients of the PAN-like group. Ozen et al. pointed out that the enzyme activity in healthy heterozygotes of their cohort was at an in-between level, while it was way below normal in DADA2 patients with one ADA2 mutation. Therefore, they suggest that siblings of patients with DADA2 take an enzyme activity test first [10]. We cannot apply this suggestion due to the unavailability of the test in our country, and the difficulty and high cost of sending the specimen to be tested abroad. Considering the fact that most of our patients had affected first-degree family members, we strongly recommend that the families of DADA2 patients are investigated for signs and symptoms of DADA2. Furthermore, we suggest genetic testing for all the first-degree members of families when they can afford the cost.

Debates are still made on the most proper treatment for ADA2 patients. Anti-TNF drugs are introduced as the drugs of choice in most cases presenting with vasculitis. They have proven potency in decreasing rates of stroke occurrence and levels of inflammatory markers. On the other hand, patients with hematologic abnormalities do not respond well to this treatment. Most PRCA and BMF patients treated with biologic agents have been dependent on transfusions. These patients can benefit from HSCT [16, 20]. The efficacy of other treatments, such as steroids, cyclophosphamide, rituximab, and azathioprine, though efficacious in some cases, have not been comparable to that of anti-TNFs [1, 13, 21, 22]. Our PRCA patient had been treated with various immunosuppressants and Etanercept was prescribed when DADA2 was diagnosed. It seems to have ceased the symptoms of vasculitis, yet the patient still needs frequent transfusions, and she is an HSCT candidate now. A novel technology of gene transfer by lentiviral vector (LV)–mediated ADA2 gene correction has recently been proposed by preclinical investigations as a potential breakthrough in the treatment of DADA2 [23, 24].

**Table 2:** Clinical features of the two groups of patients, polyarteritis nodosa (PAN)-like and Diamond-Blackfan Anemia (DBA)-like. Ig: immunoglobulin

Clinical features	PAN-like patients (10)	DBA-like patient (1)
Fever	9	Yes
Hypertension	1	No
Livedo reticularis	10	Yes
Hematologic	1 (anemia)	Anemia, neutropenia
Neurologic	7	Stroke
Myalgia/arthralgia	5	Migratory arthritis
Oral aphthosis	3	Yes
Immunologic findings	1	Decreased IgA and IgG

## Conclusions

The approach to DADA2 patients is a multi-disciplinary one, and it should be well introduced to neurologists, hematologists, and immunologists as well as rheumatologists to facilitate early recognition and treatment. Anti-TNFs are potent drugs to treat DADA2 patients; however, HSCT should be considered in patients with hematologic manifestations.

## Electronic supplementary material

Below is the link to the electronic supplementary material.

Supplementary Material 1

## Data Availability

The datasets used and/or analyzed during the current study are available from the corresponding author upon reasonable request.

## Abbreviations

ADA2: Adenosine deaminase 2
ALPS: Autoimmune lymphoproliferative syndrome
ANA: Antinuclear antibody
Anti-dsDNA: Anti-double-stranded DNA
APS: Antiphospholipid
AR: Autosomal recessive
BMF: Bone marrow failure
c-ANCA: Cytoplasmic anti-neutrophil cytoplasmic antibodies
CECR1: Cat eye syndrome chromosome region, candidate 1
CRP: C-reactive protein
DADA2: Deficiency of adenosine deaminase 2
DBA: Diamond Blackfan anemia
DHR: Dihydrorhodamine
ESR: Erythrocyte sedimentation rate
GCSF: Granulocyte-colony stimulating factor
HSCT: Hemopoietic stem cell transplantation
Ig: Immunoglobulin
IVIG: Intravenous immunoglobulin
LV: Lentiviral vector
MRI: Magnetic resonance imaging
PAN: Polyarteritis nodosa
p-ANCA: Perinuclear anti-neutrophil cytoplasmic antibodies
PRCA: Pure red cell aplasia
PRES: Posterior reversible encephalopathy syndrome
SCID: Severe combined immunodeficiency
SLE: Systemic lupus erythematosus
TNF: Tumor necrosis factor
WES: Whole exome sequencing
