# Supplementary material for: A case series of ten plus one deficiency of adenosine deaminase 2 (DADA2) patients in Iran
Source: Pediatr Rheumatol Online J. 2023 Jun 13;21:55. doi: 10.1186/s12969-023-00838-3 (PMC10265890; doi:10.1186/s12969-023-00838-3)
Supplement: Supplementary file 1 — Supplementary Material 1 [file 12969_2023_838_MOESM1_ESM.docx]

Dear editor,

Thank you very much for your interest in our article and for your invaluable comments. We have addressed all the comments of the respected reviewers, and below you can find the point-by-point answer to each comment. We have also highlighted any change we made according to the comments in the article, and we are sending the revised article to you.

Many thanks,

Vahid Ziaee

Dear reviewers,

We are grateful for the precious time you paid to review our article. We have provided point-by-point answer to your invaluable comments. We hope we can address all your concerns in the best way.

Many thanks,

Vahid Ziaee

**Reviewer #1:**

1. Given the relatively small number of case series, this report has some value. However, I think it would benefit from some restructuring and highlighting that presentation may be to a variety of subspecialists with varying awareness of the condition.

Answer:

We tried to solve this by adding a part to the discussion, which will also be explained in answer to a next comment:

Considering the manifestations and the differential diagnoses, DADA2 is not merely a rheumatologic disease, and introducing this disease to hematologists, neurologists and immunologists is mandatory to initiate prompt and proper treatment. It should be noted, however, that rheumatologic diseases can be also termed “systemic disorders”, meaning that a rheumatologist must have expertise in detecting any abnormality in any organ system. The treatment and follow-up of DADA2 patients can be managed best by a rheumatologist that should use a multidisciplinary team for help. On the other hand, rheumatologists should try to raise awareness among pediatricians, internists, hematologists and neurologists about the manifestations of this disease and the necessity of referral. A patient with stroke, for instance, should be systematically investigated by a neurologist and, if suspicious manifestations of DADA2 are found, referred to a rheumatologist as the team manager. It should be drawn into attention that the patients of this cohort were referred from doctors with different specialties or subspecialties, but the final diagnosis in all the cases was made by a rheumatologist.

2. The case presentations would be clearer if subheadings were provided for each patient number described in detail.

Answer:

We understand that this change could make our article more comprehensive; however, we had two main goals for writing this paper (as we will later explain, too): 1. To introduce the presentations of our cohort of DADA2 patients in Iran which are all of them found up to this day 2. To categorize the patients into the groups of vasculitis and hematologic manifestations, nearly similar to what Lee et al have proposed, and to compare the characteristics of these two groups. To elaborate our first goal, we should explain that this article is an epidemiologic report of all DADA2 cases of Iran, and we still need to follow the patients and observe the outcomes.

3. The reason for presenting two patients in detail should be highlighted to contextualise the subsequent paragraphs (e.g. we describe two patients in detail because they had X/Y features of interest or presented to X/Y specialists initially)

Answer:

We made the necessary edition. We added this part to the case presentation where we write about the patient who passed away:

Since this patient was our only patient who, unfortunately, passed away, we discussed the characteristics in detail in a try to understand the possible difference with other patients that could have led to the mortality.

And we added this part to discussion where we explain about the patient with PRCA:

In this case series, we depicted the characteristics of our patient with PRCA phenotype in more details, since we meant to point out the differences between DADA2 patients with mere vasculitis features and those who carry hematologic abnormalities.

4. The conclusion states that other specialists should be made aware of this condition. That could be highlighted more clearly in the introduction in order to encourage other readers.

Answer:

We added this part to introduction:

Considering the diverse manifestations, a DADA2 patient might first be referred to a neurologist, hematologist, immunologist, or rheumatologist. That is why increased awareness of the characteristics of this disease is of utmost importance.

5. It also raises the question of whether a paediatric rheumatology journal is the most appropriate place to publish the case series if the purpose is to raise awareness among specialists who may fall outside the readership of this journal.

Answer:

Although we emphasize that awareness should be raised within specialties such as hematology and neurology about DADA2, we still believe that the patients should be referred to a rheumatologist as the main care giver of the patient. We added this part to the discussion and explained more:

Considering the manifestations and the differential diagnoses, DADA2 is not merely a rheumatologic disease, and introducing this disease to hematologists, neurologists and immunologists is mandatory to initiate prompt and proper treatment. It should be noted, however, that rheumatologic diseases can be also termed “systemic disorders”, meaning that a rheumatologist must have expertise in detecting any abnormality in any organ system. The treatment and follow-up of DADA2 patients can be managed best by a rheumatologist that should use a multidisciplinary team for help. On the other hand, rheumatologists should try to raise awareness among pediatricians, internists, hematologists and neurologists about the manifestations of this disease and the necessity of referral. A patient with stroke, for instance, should be systematically investigated by the neurologist and, if suspicious manifestations of DADA2 are found, referred to a rheumatologist as the team manager. It should be drawn into attention that the patients of this cohort were referred from doctors with different specialties or subspecialties, but the final diagnosis in all the cases was made by a rheumatologist.

6. There are a few abbreviations which are not explained on first use (e.g. DHR line 100, PAN line 113)

Answer: We appreciate this comment, and we edited the abbreviations, and sorted them alphabetically.

7. There are a few typos and sentences which require restructuring (e.g. pH written as "PH" line 69, "presented *with* decreased…" line 93

Answer:

We addressed these problems. Moreover, we had a fluent English speaker edit the whole article.

8. The mutation in line 103-104 would be more clearly presented as p.G321E with the chromosomal position and RSID in brackets afterwards.

Answer:

We changed that part as follows, and we hope we have addressed the reviewer’s point correctly:

At this stage, whole exome sequencing (WES) results were prepared, showing an autosomal recessive (AR) homozygous mutation in ADA2: p.G321E [rs865858930].

9. Patient 1 had a sibling and father with the same homozygous mutation as mentioned in line 119/120; it would be informative to state if they were symptomatic and whether they were included in this case series

Answer:

Yes, they were included (patient 2 and 11). The explanations for patient 2 are included in the case presentations where we talk about treatments (we added “patient 2” there) and also in Table 1, and for patient 11 in table 1. We added this after the case presentation of patient 1 to clarify:

…was found in the brother and father (patients 2 and 11).

10. Normal ranges should be included in line 112 for the immunoglobulin levels

Answer:

This part was edited:

Antiphospholipid (APS) IgM antibody level was 2 g/L (normal range: 0.4 – 2 g/L), and IgG level was 67 g/L (normal range: 6 – 16 g/L).

The fact that CECR1 is now called ADA2 should be included in the introduction rather than first mentioned in the discussion

Answer:

We omitted it from discussion and changed the introduction this way:

CECR1 gene, now known as ADA2 gene, is mapped to chromosome 22q11.1.

11. If the patients are to be described within two groups, this should be included earlier because it is confusing to have two different tables with clinical characteristics in two different sections of the paper; I think it would make more sense to have the two tables combined because almost all the information from table 2 is included in table 1. The differences could then be discussed in more detail within the discussion section which is quite descriptive with some repetition of what was seen in the results section.

Answer:

We should explain that our cohort has two main goals: 1. To introduce the presentations of our cohort of DADA2 patients in Iran which are all of them found up to this day 2. To categorize the patients into the groups of vasculitis and hematologic manifestations, nearly similar to what Lee et al have proposed, and to compare the characteristics of these two groups. Table 2 is put to emphasize on this comparison. To elaborate more our second goal, we edited the following part in discussion:

Lee et al suggested a categorization of DADA2 patients into three major groups: vasculitis, bone marrow failure (BMF), and PRCA(15). They performed a literature review and found 100 cases of vasculitis, the most common presentation of DADA2, and 52 cases of BMF or PRCA. They also presented an international cohort of five PRCA and ten BMF patients. Lee et al found major differences between the three groups. Analogous to interpretations of their cohort and also literature review, we reported one patient with PRCA phenotype. The number of DADA2 cases with hematologic abnormalities reported in the literature is much less than those with vasculitis, and we also found only one patient among 11 patients with this phenotype. Similar to most of the PRCA patients reported in the case series, our patient’s symptoms developed very early at the age of 4 months. Her initial presentation was cytopenia and she later developed vasculitis in form of a stroke episode (15-17). In this case series, we depicted the characteristics of our patient with PRCA phenotype in more details, since we meant to point out the differences between DADA2 patients with mere vasculitis features and those who carry hematologic abnormalities. We divided our patients into two groups of PAN-like patients and DBA-like patients, close to what LEE et al have performed. The clinical characteristics of our two groups of patients are summarized in Table 2.

Moreover, we strengthened the discussion by emphasizing on the difference between the genotypes of these two groups:

All the patients in the PAN-like group had G47R mutation, and the one DBA-like patient had a p.Gly321Glu (P.G321E) mutation, located on the catalytic domain, resonating with the finding of Ozen et al. This genotype-phenotype correlation has convinced us to name our cohort as “ten plus one DADA2 patients”.

12. While the conclusion is reasonable, I think the reader needs to be led to it more clearly from the outset. I think this can be achieved but will require some reworking.

Answer:

We appreciate this comment. With the changes that we made to discussion; we hope that this problem is resolved now:

Considering the manifestations and the differential diagnoses, DADA2 is not merely a rheumatologic disease, and introducing this disease to hematologists, neurologists and immunologists is mandatory to initiate prompt and proper treatment. It should be noted, however, that rheumatologic diseases can be also termed “systemic disorders”, meaning that a rheumatologist must have expertise in detecting any abnormality in any organ system. The treatment and follow-up of DADA2 patients can be managed best by a rheumatologist that should use a multidisciplinary team for help. On the other hand, rheumatologists should try to raise awareness among pediatricians, internists, hematologists and neurologists about the manifestations of this disease and the necessity of referral. A patient with stroke, for instance, should be systematically investigated by the neurologist and, if suspicious manifestations of DADA2 are found, referred to a rheumatologist as the team manager.

**Reviewer #2**: This is an interesting article on a cohort of Iranian patients with DADA2. A few things need to be addressed. (

1)The language is stilted in many sections and many the's are left out of the sentences on pages 6 and 8, which would help clarity.

Answer:

We appreciate the comment had a fluent English speaker revise the manuscript. The changes are highlighted in the document.

(2) Many of the sentences in the manuscript are too long and need to shortened throughout the manuscript. Possibly have a person more familiar with the English language to review.

Answer:

We appreciate the comment had a fluent English speaker revise the manuscript. The changes are highlighted in the document.

(3) Figure 1 could be eliminated. Most Physicians know what livedo presents.

Answer:

The picture is a typical picture of livedo racemose, and we thought it would be good to share one for non-experts to help in differentiating between livedo reticularis and racemose. However, if the editor desires, the image can be omitted.

(4) The references need to be reviewed. Typing errors are noted, such as 18,19, and 23.

Answer:

The references are made by ENDNOTE. There might be typos in the original titles, but since we need to keep the titles of the articles in the way they are published, we have no choice but to keep them this way.

**Reviewer #3**: The authors report a series of 11 cases of ADA2 deficiency. However, the description of this cohort remains poor and poorly structured, especially in terms of biology (ESR, CRP, Ig determination at diagnosis...).

Answer:

We edited the case presentation part and added the following highlighted parts:

We introduce eleven patients with DADA2 diagnosis including one brother and sister (patients seven and six), one set of twin sisters (patients four and five), and one father and his daughter and son (patients eleven, one, and two, respectively).

Seven patients (63%) were females. The range for age of onset was 0.5 to 13 years, median=3 years. It is noteworthy that we did not know the exact age of onset of one of the patients who was an adult at the time of recruitment, but he expressed that the episodes had begun since childhood. The range for age of diagnosis was 3 to 37 years, median=8 years. Ten patients (91%) had consanguineous parents. All the patients manifested livedo racemosa/reticularis. Ten patients (91%) reported febrile episodes, and seven (64%) had experienced strokes. Only one patient had hypertension. Nine patients (81%) had elevated inflammatory markers (erythrocyte sedimentation rate (ESR) and C-reactive protein (CRP)) when they were admitted to the hospital. Two the patients (11%) presented with decreased immunoglobulin levels. ADA2 activity cannot be tested in Iran, and it is costly to send the specimen abroad and do the test. Therefore, it was only tested for two patients (patients 6 and 7), and the levels were zero for both. The clinical characteristics of the patients are summarized in Table 1.

The "treatment" part is not clear.

For patient 2, why leave him on COLCHICINE?

Answer:

We appreciate this comment, and edited the treatment section.

Regarding colchicine treatment:

We started treating the patient with colchicine when we did not know about the definite diagnosis and genetic testing was not ready yet. The patient responded well to the drug and he was not experiencing any more symptoms when we told the parents about the diagnosis of DADA2 and the need to initiate treatment with an anti-TNF. The parents did not accept to take the treatment and expressed that their son is symptom-free. To clarify, we changed treatment section in the following way:

The brother of this patient (patient 2), presented with episodes of fever, oral aphthosis and livedo racemosa. WES was done at the same time that it had been done for his sister, and he was diagnosed with DADA2 at the age of three. He is being treated with colchicine since the beginning of presentations and his attacks have not recurred in the last two years. Since he became symptom-free after initiation of colchicine, the parents refused to initiate an anti-TNF therapy when DADA2 was diagnosed. For the same reason, the diagnosis of DADA2 for the father (patient 11) did not change the fact that he was being treated with colchicine.

We also added some details to the table 1:

| **Duration of treatment with anti-TNF (months)** | - | - | 23 | 36 | 36 | 8 | 7 | 12 |  |  | - |
| --- | --- | --- | --- | --- | --- | --- | --- | --- | --- | --- | --- |
| **Outcome** | Demise | Favorable, no sign or symptom | Favorable, only Raynaud remained | Favorable, no sign or symptom | Favorable, no sign or symptom | Favorable, no sign or symptom | Favorable, no sign or symptom | Persistence of anemia and neutropenia | Favorable, no sign or symptom | Favorable, no sign or symptom | Favorable, no sign or symptom |

For mutations, apart from the classic G47R mutation, is the mutation of patient 8 known to cause bone marrow failure?

Answer:

Yes. This is well elaborated in the study of Ozen et al. to which we pointed in our discussion. The explanation for the mutation of patient 8 (G321E) is included in this section of the discussion:

Ozen et al surveyed the clinical and molecular differences between two groups of DADA2 patients: 14 in PAN-like group and 10 in DBA-like group. They reported that the catalytic domain of ADA2 was affected in DBA-like patients while the dimerization domain was affected in PAN-like patients(10). Similar to the study of Ozen et al, we can categorize our patients into PAN-like, who presented with vasculitis features and constitute of eight patients, and DBA-like, that is our one patient presenting with anemia and neutropenia. All the patients in the PAN-like group had G47R mutation, and the one DBA-like patient had a p.Gly321Glu (P.G321E) mutation, located on the catalytic domain, resonating with the finding of Ozen et al.

How was the diagnosis made? by neurologists? Dermatologists? or rheumatologists?

Answer:

The patients were referred form doctors with different specialties or subspecialties to the rheumatologists, and the final diagnosis was made by a rheumatologist. We added the following section to the discussion to clarify:

Considering the manifestations and the differential diagnoses, DADA2 is not merely a rheumatologic disease, and introducing this disease to hematologists, neurologists and immunologists is mandatory to initiate prompt and proper treatment. It should be noted, however, that rheumatologic diseases can be also termed as “systemic disorders”, meaning that a rheumatologist must have expertise in detecting any abnormality in any organ system. The treatment and follow-up of DADA2 patients can be managed in the best way by a rheumatologist that should use a multidisciplinary team for help. On the other hand, rheumatologists should try to raise awareness among pediatricians, internists, hematologists and neurologists about the manifestations of this disease and the necessity of referral. A patient with stroke, for instance, should be systematically investigated by the neurologist and, if suspicious manifestations of DADA2 are found, referred to a rheumatologist as the team manager. It should be drawn into attention that the patients of this cohort were referred form doctors with different specialties or subspecialties, but the final diagnosis in all the cases was made by a rheumatologist.

The tables need to be revised because they are not very clear.

Answer:

We designed table 1 based on the tables of patient characteristics in similar articles working on DADA2 patients. The references are the following:

1. Nanthapisal, S., Murphy, C., Omoyinmi, E., Hong, Y., Standing, A., Berg, S., . . . Youngstein, T. (2016). Deficiency of adenosine deaminase type 2 (DADA2).

2. Caorsi, R., Penco, F., Schena, F., & Gattorno, M. (2016). Monogenic polyarteritis: the lesson of ADA2 deficiency. Pediatric Rheumatology, 14(1), 1-16

3. Schepp, J., Bulashevska, A., Mannhardt-Laakmann, W., Cao, H., Yang, F., Seidl, M., . . . Grimbacher, B. (2016). Deficiency of adenosine deaminase 2 causes antibody deficiency. Journal of Clinical Immunology, 36(3), 179-186.

However, we tried to simplify the table by omitting ADA2 activity, and mentioning it within the text:

ADA2 activity cannot be tested in Iran and it is very costly to send the specimen abroad and do the test. Therefore, it was only tested for two patients (patient 6 and 7), and the levels were zero for both.

We also added one row to table 1 and edited one other row:

| **Duration of treatment with anti-TNF (months)** | - | - | 23 | 36 | 36 | 8 | 7 | 12 |  |  | - |
| --- | --- | --- | --- | --- | --- | --- | --- | --- | --- | --- | --- |
| **Outcome** | Demise | Favorable, no sign or symptom | Favorable, only Raynaud remained | Favorable, no sign or symptom | Favorable, no sign or symptom | Favorable, no sign or symptom | Favorable, no sign or symptom | Persistence of anemia and neutropenia | Favorable, no sign or symptom | Favorable, no sign or symptom | Favorable, no sign or symptom |

Regarding table 2, we clarified the reasoning behind the table by adding the following highlighted section to the discussion:

Lee et al suggested a categorization of DADA2 patients into three major groups: vasculitis, bone marrow failure (BMF), and PRCA (15). They performed a literature review and found 100 cases of vasculitis, the most common presentation of DADA2, and 52 cases of BMF or PRCA. They also presented an international cohort of five PRCA and ten BMF patients. Lee et al found major differences between the three groups. Analogous to interpretations of their cohort and also literature review, we reported one patient with PRCA phenotype. The number of DADA2 cases with hematologic abnormalities reported in the literature is much less than those with vasculitis, and we also found only one patient among 11 patients with this phenotype. Similar to most of the PRCA patients reported in the case series, our patient’s symptoms developed very early at the age of 4 months. Her initial presentation was cytopenia and she later developed vasculitis in form of a stroke episode (15-17). In this case series, we depicted the characteristics of our patient with PRCA phenotype in more details, since we meant to point out the differences between DADA2 patients with mere vasculitis features and those who carry hematologic abnormalities. We divided our patients into two groups of PAN-like patients and DBA-like patients, close to what LEE et al have performed. The clinical characteristics of our two groups of patients are summarized in Table 2.

**Reviewer #4:** This paper presents a valuable case series on DADA2 patients in Iran.

While the paper does not contribute any novel findings to the existing literature, it is important to have data from different national cohorts to be compared with various regions in the future, given the rarity of the disease.

1. As the main aim of anti-TNF therapy is to prevent stroke, which can occur at any time and is unpredictable, the use of only colchicine for treating two patients (Pt. 2 and 11) appears to be risky. It would be helpful if the authors could provide further comments on this matter.

Answer:

We added the highlighted parts to the discussion to clarify:

The brother of this patient (patient 2), presented with episodes of fever, oral aphthosis and livedo racemosa. WES was done at the same time that it had been done for his sister, and he was diagnosed with DADA2 at the age of three. He is being treated with colchicine since the beginning of presentations and his attacks have not recurred in the last two years. Since he became symptom-free after initiation of colchicine, the parents refused to initiate an anti-TNF therapy when DADA2 was diagnosed. For the same reason, the diagnosis of DADA2 for the father (patient 11) did not change the fact that he was being treated with colchicine.

2. To enhance the quality of the paper, a systematic review of the patients reported in the literature could be included. However, this may require extending the manuscript from a case report to a systematic review, and the authors' willingness to do so is unknown.

Answer:

We appreciate this comment, and we understand that a review of the literature can improve the quality of this paper; however, we had two main goals for writing this paper (as we will later explain, too): 1. To introduce the presentations of our cohort of DADA2 patients in Iran which are all of them found up to this day 2. To categorize the patients into the groups of vasculitis and hematologic manifestations, nearly similar to what Lee et al have proposed, and to compare the characteristics of these two groups. We think that it will be better if a separate systematic review on DADA2 patients is published.

3. Additionally, the paper requires significant professional English editing as sentences are either overly lengthy or convoluted, making it difficult to follow.

Answer:

We appreciate this comment, and had a fluent English speaker revise the manuscript.

**Reviewer #5:** Excellent series of 11 patients with DADA2. The table is especially effective.

A few minor questions:

1. What was the etiology of the patient's hypertension? Was renal artery stenosis present?

Answer:

The abdominal MR angiography showed suspicious stenosis in the renal artery. After treatment, hypertension was resolved.

2. The term "auto-inflammatory" is not present in the abstract, and DADA2 deficiency is not identified as an autoinflammatory disease until paragraph 3 of the introduction. I would encourage including the term Auto-inflammatory in the beginning of the abstract.

Answer:

We added the term “autoinflammatory” to the beginning of background part in the abstract, and to the beginning of introduction:

**Background**

Deficiency of adenosine deaminase 2 (DADA2) is an autosomal recessive autoinflammatory disease…

**Introduction**

The autoinflammatory disorder of the deficiency of adenosine deaminase 2 (ADA2) or DADA2…

3. The description of DADA2 def patients as having a "wide spectrum of clinical presentations" is not supported by your detailed descriptions of these patients who presented with fevers, vasculitic skin lesions = livedo racemosa, and most with stroke. It would strengthen your presentation of these cases - which as you advocate are extremely important for neurology, hematology and immunology, in addition to General Pediatricians, to recognize - if you emphasize the common features of this disease.

Answer:

We tried to resolve this issue by elaborating more in two sections of the discussion:

DADA2 patients can present with a wide range of manifestations. Episodes of fever and systemic symptoms, vasculitis features such as the notable sign of recurrent strokes, skin manifestations and most commonly livedo racemosa/reticularis, hypertension, hematologic manifestations and immunologic dysregulations are among the most common signs and symptoms. Besides the mentioned symptoms, our patients manifested with oral ulcers, genital ulcers, myalgia, and headache, which are also reported in the literature, albeit less commonly (10, 12-14).

….

Moreover, characteristics of immunodeficiencies in terms of cell depletions and antibody deficiencies can be found in DADA2 patients(18). Considering the manifestations and the differential diagnoses, DADA2 is not merely a rheumatologic disease, and introducing this disease to hematologists, neurologists and immunologists is mandatory to initiate prompt and proper treatment. It should be noted, however, that rheumatologic diseases can be also termed as “systemic disorders”, meaning that a rheumatologist must have expertise in detecting any abnormality in any organ system. The treatment and follow-up of DADA2 patients can be managed in the best way by a rheumatologist that should use a multidisciplinary team for help. On the other hand, rheumatologists should try to raise awareness among pediatricians, internists, hematologists and neurologists about the manifestations of this disease and the necessity of referral. A patient with stroke, for instance, should be systematically investigated by the neurologist and, if suspicious manifestations of DADA2 are found, referred to a rheumatologist as the team manager. It should be drawn into attention that the patients of this cohort were referred form doctors with different specialties or subspecialties, but the final diagnosis in all the cases was made by a rheumatologist.

Reviewer #6: Thank you for your work.

+ interesting cohort of DADA2 patients

+ abstract is well written and informative.

Content:

1. most DADA2 patients are compound heterozygous. or homozygous. Pt. 6+7 are heterozygous. Could you discuss that?

Answer:

We added this section to the discussion:

The mutations found in DADA patients are less commonly heterozygous. However, no correlation has been found between heterozygosity and the phenotype(10, 13). Likewise, our two patients with heterozygous mutations of G47R, who were brother and sister, did not show distinct manifestations in comparison with the rest of the patients of the PAN-like group. Ozen et al. pointed out that the enzyme activity in healthy heterozygotes of their cohort was at an in-between level, while it was way below normal in DADA2 patients with one ADA2 mutation. Therefore, they suggest that siblings of patients with DADA2 take an enzyme activity test first(10). We cannot apply this suggestion due to the unavailability of the test in our country, and the difficulty and high cost of sending the specimen to be tested abroad. Considering the fact that most of our patients had affected first-degree family members, we strongly recommend that the families of DADA2 patients are investigated for signs and symptoms of DADA2. Furthermore, we suggest genetic testing for all the first-degree members of families when they can afford the cost.

2. unclear reference line 144/145- do you compare your cohort to the Lee et al cohort? or just one patient?

Answer:

Lee et al made a categorization for DADA2 patients: BMF, DBA, and vasculitis. We have done nearly the same categorization. Since the number of patients with hematologic manifestations is much less than those with vasculitis, in our cohort of 11 patients we had one with DBA-like presentation. We edited the explanation in the article to clarify:

Lee et al suggested a categorization of DADA2 patients into three major groups: vasculitis, bone marrow failure (BMF), and PRCA(15). They performed a literature review and found 100 cases of vasculitis, the most common presentation of DADA2, and 52 cases of BMF or PRCA. They also presented an international cohort of five PRCA and ten BMF patients. Lee et al found major differences between the three groups. Analogous to interpretations of their cohort and also literature review, we reported one patient with PRCA phenotype. The number of DADA2 cases with hematologic abnormalities reported in the literature is much less than those with vasculitis, and we also found only one patient among 11 patients with this phenotype. Similar to most of the PRCA patients reported in the case series, our patient’s symptoms developed very early at the age of 4 months. Her initial presentation was cytopenia and she later developed vasculitis in form of a stroke episode(15-17). In this case series, we depicted the characteristics of our patient with PRCA phenotype in more details, since we meant to point out the differences between DADA2 patients with mere vasculitis features and those who carry hematologic abnormalities. We divided our patients into two groups of PAN-like patients and DBA-like patients, close to what LEE et al have performed. The clinical characteristics of our two groups of patients are summarized in Table 2.

Definition of outcome is lacking (Table 1), inactive disease?

Answer:

We added the description to the table:

| **Outcome** | Demise | Favorable, no sign or symptom | Favorable, only Raynaud remained | Favorable, no sign or symptom | Favorable, no sign or symptom | Favorable, no sign or symptom | Favorable, no sign or symptom | Persistence of anemia and neutropenia | Favorable, no sign or symptom | Favorable, no sign or symptom | Favorable, no sign or symptom |
| --- | --- | --- | --- | --- | --- | --- | --- | --- | --- | --- | --- |

lines 166 ff: not in discussion, rather description of the cohort/patients

Answer:

We deleted this part.

line171: HSCT: could you mention your criteria for HSCT? Transfusions, etc. Do you have any experience?

Answer:

Although we have good experience in HSCT of patients with inborn errors of immunity, we do not have any experience in HSCT of DADA2 patients. Our PRCA patient is our first DADA2 patient who is a candidate for HSCT. As we mentioned in the article with references, HSCT seems to be a good choice for DADA2 patients with hematologic manifestations especially when anti-TNFs are not useful. Likewise, our PRCA patient needed continuous transfusions in spite of anti-TNF therapy. That is why we made her a candidate of HSCT.

Please distinguish clearly between patient characteristics and discussion.

Answer:

We deleted irrelevant parts from discussion.

Highlight your genotype phenotype correlation- that is interesting.

Answer:

We emphasized on this matter in the discussion, in the paragraph we explain about the genotype-phonotype correlation, and added the following highlighted parts:

All the patients in the PAN-like group had G47R mutation, and the one DBA-like patient had a p.Gly321Glu (P.G321E) mutation, located on the catalytic domain, resonating with the finding of Ozen et al. This genotype-phenotype correlation has convinced us to name our cohort as “ten plus one DADA2 patients”.

The mutations found in DADA patients are less commonly heterozygous. However, no correlation has been found between heterozygosity and the phenotype (10, 13). Likewise, our two patients with heterozygous mutations of G47R, who were brother and sister, did not show distinct manifestations in comparison with the rest of the patients of the PAN-like group. Ozen et al. pointed out that the enzyme activity in healthy heterozygotes of their cohort was at an in-between level, while it was way below normal in DADA2 patients with one ADA2 mutation. Therefore, they suggest that siblings of patients with DADA2 take an enzyme activity test first (10). We cannot apply this suggestion due to the unavailability of the test in our country, and the difficulty and high cost of sending the specimen to be tested abroad. Considering the fact that most of our patients had affected first-degree family members, we strongly recommend that the families of DADA2 patients are investigated for signs and symptoms of DADA2. Furthermore, we suggest genetic testing for all the first-degree members of families when they can afford the cost.

Abbreviations:

please sort by alphabet

include all abbreviations, eg. ANA, p-ANCA, c-ANCA, DHR, etc.

Answer: We appreciate this comment, edited the abbreviations, and sorted them alphabetically.

Spelling: please use consistent spelling PANCA vs. p-ANCA etc.

Answer: Done.

Units: please use correct units: eg. line 112: U/l, etc.

Answer: Done

IgM antibody level was 2 g/L (normal range: 0.4 – 2 g/L), and IgG level was 67 g/L (normal range: 6 – 16 g/L).

repetitions: the death of patient one is mentioned in line 106, 116, 124, 126 and table 1- you might find a more concise way of description of the cohort.

Answer:

We omitted the repetitions. Currently we are mentioning this demise in line 133 (presenting the case), 142 (treatment section) and in Table 1, where we have to summarize the outcome of all the patients. The reason that we mention the demise once in case description is that we only describe the characteristics of two patients in our cohort: one with PRCA, and one who was dead before initiation of anti-TNF, and that is a very important matter to emphasize on. Our treatment section is separate so we have to mention the outcome again.
